# Addressing the challenges of dementia care in Nigeria: A call for a comprehensive national strategy

**DOI:** 10.1002/puh2.186

**Published:** 2024-05-15

**Authors:** Oluwagbemiga Oyinlola

**Affiliations:** ^1^ School of Social Work McGill University Montreal Canada; ^2^ Medical Social Services Department University College Hospital Ibadan Nigeria

**Keywords:** caregivers, dementia, geriatrics, health policy, public health

## Abstract

Despite the growing prevalence of dementia, driven by an ageing population and compounded by cultural misunderstandings and stigma, Nigeria lacks a coherent national plan to address this issue. The article points out that although Nigeria has enacted policies such as the National Aging Policy, which do not sufficiently address the specific needs of people living with dementia. It underscores the necessity of integrating a dementia strategy within the broader health and social care systems of Nigeria. The article draws on the World Health Organization's Global Dementia Action Plan to elaborates on several critical areas for action, including public health prioritization of dementia, awareness and stigma reduction, improved diagnosis, treatment, care, and support, alongside bolstering support for caregivers. It stresses the importance of a dementia‐friendly infrastructure, research and innovation, and leveraging community support to foster a more inclusive society. Furthermore, the article outlines current state of healthcare and social support systems in Nigeria, pointing to significant gaps in infrastructure, healthcare workforce, and financial mechanisms that hinder effective dementia care. Hence, elevating dementia care as a national health priority, and increasing access to quality care and support, Nigeria is well positioned to mitigate the impact of dementia on families and the person with dementia. The call to action is clear: a national dementia strategy, informed by global best practices and tailored to Nigeria's unique cultural and societal context, is essential for addressing the challenges of dementia care and improving the well‐being of affected individuals and their families in Nigeria.

## INTRODUCTION

The global burden of dementia presents a significant and growing challenge, particularly in low‐ and middle‐income countries (LMICs), where resources and healthcare systems often lack the capacity to effectively address complex health issues [1, 2]. According to the World Health Organization (WHO), approximately 50 million people worldwide are living with dementia as of 2021, with nearly 60% of these individuals residing in LMICs, a figure that is expected to increase as populations age [3]. The implications of the growing dementia burden in Nigeria and similar LMICs are far‐reaching. Beyond the direct impact on the health and quality of life of individuals with dementia [4], the condition places a heavy burden on families, often requiring significant emotional, physical, and financial resources for care.

As Nigeria, the most populous nation in Africa, grapples with ageing population among its over 260 million inhabitants, and than 9.4 million aged 60 years and older [5], the challenge of dementia care has become increasingly critical. The country has a current dementia prevalence rate of 4.9% [4, 6], the nation faces multifaceted obstacles ranging from a pronounced brain drain affecting the healthcare profession [7, 8] to fragmented geriatric care services and a pervasive lack of knowledge about dementia [4, 9, 10]. These issues underscore the urgent need for a well‐coordinated and comprehensive approach to dementia care programming within the country.

In addition, dementia care is predominantly provided through informal systems, largely reliant on family and community support due to the country's strong cultural and familial ties. For example, home‐based care is ingrained in Nigerian society, where families are culturally expected to care for older adults [11, 12]. However, this can significantly burden caregivers, who often lack formal support, training, and resources. The demographic trends indicate a geometric progression in the ageing population, placing additional strain on already stretched healthcare resources and necessitating innovative strategies to address the growing demand for dementia care. Hence, adopting a more structured approach to dementia care, informed by both local cultural norms and international best practices, can help address the needs of individuals with dementia and their families more effectively.

Reflecting upon the insights the WHO provided in 2021 regarding the public health response to dementia, it becomes evident that the execution of the Global Dementia Action Plan 2017 encountered significant hurdles in LMICs [2]. These challenges were primarily attributed to inadequate infrastructural support from governmental entities and the absence of comprehensive national dementia plans.

Despite the escalating global prevalence of dementia and the growing number of individuals living with this condition, no African nation has established an integrated national dementia strategy [4, 13]. This lack of strategic planning is further compounded by the pervasive stigma associated with dementia. Such stigma contributes to a widespread misunderstanding and lack of empathy toward those affected by the condition, posing significant barriers to effective care and support [4, 14–16]. The complexity of designing and implementing dementia awareness campaigns and educational initiatives that effectively cater to the diverse populations of countries like Nigeria cannot be overstated. It requires a meticulous approach that takes into consideration the cultural specificities and sensitivities of the target population.

Nigeria recently passed the National Ageing Policy [17], though an important step toward improving health and social well‐being of older adults in Nigeria, however, did not adequately address the growing needs, rights, and justice for people with dementia and their families in the country. Further, this article calls for a plan of dementia action that is framed in line with the seven‐action area of the 2017 WHO global action plan: (a) dementia as a public health priority; (b) awareness and friendliness; (c) risk reduction; (d) dementia diagnosis, treatment, care, and support; (e) support for dementia carers; (f) information system for diagnosis; and (g) research and innovation [18] with the potential of addressing challenges, and opportunities to dementia strategy. The role of potential stakeholders in the implementation of dementia care in Nigeria was highlighted in this paper. The article aimed to explore the multifaceted challenges associated with dementia care in Nigeria, highlighting the urgent need for a comprehensive national strategy to improve the well‐being of individuals with dementia and their families.

### Absence of dementia care strategy in the national ageing policy

Nigeria is heterogeneous, with more than 350 ethnic‐linguistic groups, with major groups being Yoruba in the Southwest, Hausa in the North, and Igbo in the Southeast. These groups are similar in cultural background, especially in their cultural perception of care for older adults. Although the 1999 constitution of the Federal Republic of Nigeria insists that “suitable and adequate shelter and food, reasonable minimum living wage, old age care and pension and unemployment, sick benefits, and welfare of the disabled are provided for all citizens” (Section 16 (2)D), there are still few policies designed to care for older adults, that is no more functional [19]. For example, the National Social Development Policy of 1989 was designed to cater to the needs of vulnerable people in Nigeria, which captured older adults who were classified as vulnerable, and it was last implemented during the military regime in 1996, but nothing has been done to reactivate the policy [20]. Despite this cultural homogeneity in valuing older adults, Nigeria faces a glaring absence of a coherent national dementia plan or policies specifically tailored toward the comprehensive care of its aging population. This oversight undermines the cultural and constitutional mandates of older adults and signals a critical need for a national dementia plan.

Nigeria witnessed a significant milestone in ageing programming through the enactment of the National Senior Citizen Centers (NSCC) Act, which provides for the needs of Nigerians aged 70 [21]. This center was approved for implementation in 2021, with the mandate that senior citizen centes should be established across 36 states in Nigeria [21], including the identification of needs of older adults, training and opportunities for senior citizens in the country through the creation of sports, educational, health, and social programming for older adults in the country [21].

In addition, the National Senior Citizens Center's mandates are to create and implement programs that help Nigerian older adults find work or secure income, access health and professional services, and keep statistics of Nigerian older adults [21]. The establishment of NSCC has birthed a series of innovative programs and action plans compliant with global standards of care and support for older adults. However, the impact of the NSCC activities could only be felt at the federal level, whereas implementation in state regions has suffered significant setbacks and poor implementation outcomes.

Consequently, until 2021, Nigeria did not have a functional national policy on older adults, the government of Nigeria started the drafting and development of a national policy on aging in 2008, but nothing was done, even not until 2018, the new administration of the Federal Ministry of Humanitarian Affairs and the Ministry of Health partnered with the WHO and other development partners in the country to develop a National Policy on Aging, which was ratified by the government of Nigeria in 2021 [22]. The primary goal of the policy was to promote laws that protect the rights and privileges of Nigerian older adults, enhance their quality of life, strengthen the traditional support institution for the care of older adults, ensure sustainable income, guarantee their fundamental human rights, and create data management for effective planning, research, and evaluation of aging programming in Nigeria [22].

The Nigerian National Policy on Aging also applies the regional and international instruments domesticated in Nigeria, such as the African Union Policy Framework and the Plan of Action on Aging (2002); the United Nations Universal Declaration of Human Rights (1948), the UN International Plan of Action on Aging, the UN Political Declaration on Aging (2002), and the Sustainable Development Goals (SDGs) [23]. Despite these landmark achievements, none of the policy's objectives considered the needs of older adults living with dementia and their caregivers in Nigeria. In addition, most states in Nigeria are still grabbing with the domestication of the policy to benefit older adults.

Although the Nigeria National Policy on Aging is guided by the Madrid International Plan of Action on Aging, the WHO global strategies on health and aging, including the African charter human rights of older people and SDGs Agenda of 2030, have the important role of institutions in supporting older adults, especially in addressing all forms of stereotypes associated with aging, including those living with all forms of disabilities (e.g., dementia and other neurocognitive disabilities). Nigeria's commitment to international agreements such as the Madrid International Plan of Action on Aging [24], WHO global strategies on health and aging [25], the African Charter on Human Rights of Older People [26], and the SDGs Agenda of 2030 [27] underscores the stereotypes associated with aging other than dementia. Hence, a dedicated dementia strategy is essential for addressing stereotypes associated with aging.

Though promotion of awareness and understanding of dementia, the strategy can contribute to dispelling myths and misconceptions surrounding cognitive disabilities, fostering a more inclusive and supportive society. Furthermore, there is a plethora of media portrayals of people living with dementia as demeaning, socially excluded, and spiritually possessed [28, 29]. Beyond the individual, dementia has far‐reaching implications for families, communities, and the nation as a whole. Despite this, the rights and privileges of this population of older adults are not recognized and acknowledged in the policy documents. Therefore, the call for a National Dementia strategy in Nigeria is not just a matter of policy; it is a collective moral obligation to protect the dignity and well‐being of our fellow citizens facing this condition.

### Nigeria's preparedness for a national dementia strategy

Dementia research and its ethnic disparities in Nigeria have not gained extensive documentation as in more developed countries. However, the prevalence of dementia can be influenced by a variety of factors including genetic, environmental, and lifestyle factors that vary among different ethnic groups [6]. Given Nigeria's vast ethnic diversity, it is plausible that some groups may exhibit higher or lower prevalence rates due to a combination of these factors, though specific, large‐scale studies pinpointing particular ethnic groups with higher dementia prevalence within Nigeria are limited.

For example, a comprehensive analysis of dementia in Nigeria reported a pooled crude prevalence rate of 4.9%, with a notably higher occurrence in women (6.7%) compared to men (3.1%) [30]. The study, encompassing 43 data points, showed considerable variation across different regions and types of dementia [30]. The prevalence rates for dementia subtypes were notably lower than the overall dementia rate, with Alzheimer's disease being the most common at 2.0% [6, 14]. The study also highlights a significant increase in dementia prevalence with age, peaking at 11.0% among individuals aged 90 years or older. Additionally, urban areas reported a higher prevalence (4.9%) compared to rural settings (2.8%), underscoring the impact of geographical and lifestyle factors on dementia rates in Nigeria [14]. High heterogeneity among studies (*I* ^2^ = 98.8%, *p* < 0.001) indicates diverse population dynamics and methodological approaches in the research conducted [4].

The surge in dementia cases by 400% from 1995 to 2015 among those aged 60+ highlights a growing public health challenge [4]. A strategic imperative emerges for Nigeria, necessitating an encompassing and lucid national dementia plan. Despite sporadic advocacy endeavors from select organizations within the African region, a mere quarter of countries in Africa have devised a national policy, strategy, or plan catering to the exigencies of individuals afflicted with dementia and their familial support structures. The development of national dementia strategies in countries with similar resource settings as Nigeria offers valuable lessons and models for crafting effective approaches to dementia care.

For example: Tanzania has been pioneering the implementation of community‐based dementia care models that recognize the importance of local understanding and cultural sensitivity in dementia care [31]. The country has focused on training community health workers to identify dementia symptoms and provide basic support. Similarly, South Africa has made strides in integrating dementia care into its primary healthcare system. The country's aging policy emphasizes training primary healthcare workers in recognizing and managing dementia, ensuring that individuals with dementia receive timely and appropriate care [32, 33]. South Africa's approach demonstrates the importance of leveraging existing healthcare infrastructure to address dementia, making care more accessible and reducing the need for specialized dementia centers that may be scarce in resource‐limited settings. Moreover, there are training programs for caregivers in Kenya, providing them with the knowledge and skills needed to care for individuals with dementia effectively [34].

Additionally, Kenya has established support groups and networks for caregivers, offering them a platform for sharing experiences and accessing psychological support [34, 35]. The focus on caregivers not only improves the quality of care for individuals with dementia but also addresses the emotional and physical well‐being of caregivers themselves. Drawing from these examples, Nigeria can formulate a national dementia strategy that incorporates the training of community health workers and leverages local networks to improve access to care and reduce stigma. Integration of dementia care into the primary healthcare system ensures widespread access and efficient use of resources. Nigeria can utilize technology to enhance awareness, education, and remote support, particularly in underserved areas. The absence of a cohesive and comprehensive approach underscores the exigency for Nigeria to navigate this impending healthcare challenge with foresight and precision.

#### Awareness and stigma

The existing empirical evidence on dementia awareness within sub‐Saharan African countries underscores a substantial knowledge gap, with awareness levels varying across populations [1, 36, 37]. Notably, in Nigeria, elucidation of behaviors related to individuals with dementia often attributes such manifestations to supernatural phenomena, such as witchcraft and evil spirits, prompting recourse to traditional healing practices [15, 29]. Furthermore, the lack of comprehension regarding dementia signs and symptoms among family members contributes to delayed diagnoses and inadequate support for affected individuals and their families [4, 28].

The prevalent misconception of dementia as a natural facet of aging perpetuates stigmatization in numerous regions of Nigeria [28]. Cultural beliefs and superstitions additionally fuel misconceptions about the origins and repercussions of dementia in the Nigerian context. The resultant stigma manifests in fear and isolation, extending to avoidance behaviors even among healthcare professionals [38, 39]. These observations underscore the imperative of an ongoing collaborative effort involving government agencies, healthcare professionals, community leaders, and advocacy groups to address and mitigate awareness gaps and stigma associated with dementia.

#### Family caregivers

The caregiving landscape for older adults in Nigeria is intricately woven within multigenerational living arrangements and kinship family systems [11]. As the severity of dementia symptoms escalates and dependence on family caregivers intensifies, there is a notable trend of seeking diverse forms of assistance, a phenomenon that did not receive adequate attention in empirical literature [40]. Existing research suggests that family members typically resort to orthodox care facilities only in the advanced stages of the disease, a practice that has become normative and is not necessarily linked to a lack of awareness about dementia, even within policymaking circles [41].

The reluctance of family members to seek orthodox care arises from deep‐rooted beliefs that dementia is a curse from ancestors and a supernatural affliction, rendering it impervious to conventional medical interventions. This perception contributes to the stigma and embarrassment associated with the atypical behaviour of individuals with dementia, further exacerbated by the fear that community residents may attribute witchcraft to those afflicted, resulting in potential harm, such as stoning incidents [28]. Remarkably, a study among 10 religious ministers revealed that dementia is attributed to evil spirits, advocating for spiritual prayers and traditional concoctions as the most appropriate forms of treatment [40]. Consequently, family members often invest substantial financial resources in alternative care modalities, including sacrifices and frequent visits to religious institutions. It is crucial to note that such practices, while prevalent, lack demonstrable clinical and economic benefits and are not endorsed by clinicians as recommended care for individuals with dementia. This underscores the need for a nuanced understanding of cultural and belief systems influencing caregiving decisions while concurrently promoting evidence‐based interventions for dementia care.

#### Health infrastructures

The provision of services for individuals with dementia in Nigeria is characterized by fragmentation, primarily concentrated at the tertiary level of healthcare, thereby remaining distant from the accessibility of the average older adult [42]. Complex bureaucratic impediments further hinder potential clients from accessing follow‐up care and treatment, exacerbating the challenges in the existing healthcare framework. Despite the availability of diagnostic services, such as scans, in most tertiary healthcare facilities, and an increasing availability of psychotropic drugs, the financial burden associated with these services poses a significant challenge for the average Nigerian. The practice of out‐of‐pocket payments for healthcare exacerbates the precarious circumstances faced by individuals with dementia and their families, navigating a landscape of diverse and undocumented sources for treatment and care. It is worth noting that, as of the composition of this article, there is an absence of dedicated health insurance schemes tailored for older adults in the country.

The National Health Insurance Scheme (NHIS) is Nigeria's primary health insurance scheme. However, its coverage for older adults is limited. The NHIS typically covers formal sector workers and their dependents, leaving out a significant portion of the elderly population who are not formally employed or are in the informal sector. Moreover, the NHIS has been criticized for its low coverage rates and challenges in accessing care, particularly for specialized services like dementia care [43]. Without dedicated health insurance schemes tailored for older adults and comprehensive coverage for conditions like dementia, individuals and families affected by dementia continue to bear a substantial financial burden. This absence not only underscores the existing gaps in the healthcare system but also renders developing and implementing a national dementia strategy seemingly unrealistic within the current healthcare infrastructure. The need for comprehensive reforms, both in terms of service accessibility and financial support mechanisms, is imperative to address the burgeoning challenges associated with dementia care in Nigeria.

#### Agency efforts

Acknowledging the government's commendable efforts in establishing standalone geriatric health facilities in Nigeria, it is crucial to note that the current count reveals only two such centers, both situated in southwestern Nigeria. This limited infrastructure attempts to address the healthcare needs of a substantial population exceeding 10 million Nigerian older adults. These facilities, namely, the Chief Tony Anenih Geriatric Center at the University College Hospital, Ibadan (established in 2012), and the Ilesha Geriatric Center at Obafemi Awolowo University Teaching Hospital, Ilesha, Osun State (established in 2022), operate at the tertiary level of healthcare.

Despite their commendable efforts, their impact is constrained by their geographical concentration. Some specific initiatives from these geriatric centers include providing services to Nigerians, including a dementia clinics that offer multidisciplinary care facilitated by a team of professionals, including family physicians, neurologists, physiotherapy psychiatrists, nurses, and social workers [9, 19]. However, the reach of such centers is limited, and access can be challenging, especially for those living in rural areas. While contributing to enhancing the quality of life for a select number of older adults in Nigeria, these institutions face a critical challenge—the emigration of many experts to opportunities abroad [7]. This, in turn, poses a significant obstacle to the local capacity for dementia care, impeding research and development initiatives, and slowing progress in understanding and addressing the intricate challenges associated with dementia within the Nigerian context. Efforts to retain and incentivize local expertise are pivotal to fortifying the foundation for comprehensive dementia care and research in Nigeria.

Given the anticipated rise in the population of older adults living with dementia, it is imperative for the government and stakeholders in Nigeria to proactively prepare for the impending challenges and complexities associated with caring for this demographic and their families in the near future. The WHO's global action plan on the public health response to dementia (2017–2025) presents seven strategic actions designed to tackle challenges, harness opportunities, and provide recommendations. Importantly, it delineates clear roles for various stakeholders in the formulation and execution of a national dementia strategy tailored to the Nigerian context. Embracing and implementing such a strategy will be instrumental in mitigating the impact of dementia, enhancing care provisions, and fostering a comprehensive and sustainable approach to address the evolving needs of older adults living with dementia and their families in Nigeria.

### Challenges of implementing a National Dementia Action Plan in Nigeria

The imperative to implement a National Dementia Action Plan is clear, and the path is fraught with obstacles. Resource constraints, infrastructural limitations, and cultural barriers significantly hinder the development and execution of such a plan. Understanding these challenges is the first step toward creating a viable and effective response to the rising tide of dementia cases in the country.

#### Resource constraints

One of the most formidable challenges in implementing a National Dementia Action Plan in Nigeria is the acute shortage of financial, human, and medical resources [4]. Nigeria's healthcare funding has not improved, as it is significantly lower than the global average (54), severely limiting the availability of dementia‐related services and care. The country also faces a critical shortage of trained healthcare professionals specialized in geriatrics and neurology [41]. Moreover, the allocation of the available resources often prioritizes infectious diseases over noncommunicable conditions like dementia, reflecting a gap in the national health policy's focus. The scarcity of diagnostic tools and medication further exacerbates the challenge, making it difficult to provide comprehensive care to dementia patients [41].

#### Infrastructural limitations

Nigeria's healthcare infrastructure is not adequately equipped to support the needs of individuals with dementia. Most healthcare facilities are concentrated in urban areas, leaving over forty percent in rural populations underserved [4]. This geographical disparity in healthcare access is a significant hurdle for a National Dementia Action Plan, as it restricts the reach of dementia care services. Furthermore, existing health facilities often lack the necessary equipment and environment conducive to care  for dementia patients, such as safe, navigable spaces and specialized care units. The absence of a national data management system for dementia also poses a challenge, hindering effective monitoring, planning, and implementation of dementia care strategies.

#### Cultural barriers

Cultural perceptions of dementia in Nigeria often attribute the symptoms to supernatural causes or stigmatize those affected, leading to social isolation and neglect [28]. Such beliefs can discourage families from seeking medical care for their loved ones, preferring traditional healers or keeping the condition hidden. This cultural stigma around dementia complicates early diagnosis and treatment, making community‐based awareness and education programs essential components of a Dementia Action Plan. Moreover, the strong emphasis on family care in Nigerian culture, while beneficial in many respects, can place an undue burden on family members who lack the knowledge and resources to provide appropriate care.

#### Familiar caregivers’ challenges

One of the primary challenges is the emotional toll of watching a loved one's cognitive decline, which can lead to significant stress, anxiety, and depression [37]. For families in Nigeria, they often experience feelings of grief for the loss of the person they once knew, coupled with constant worry about the care recipient's safety and well‐being. Physically, the demands of caregiving can be exhausting, as it often requires round‐the‐clock attention, leaving little time for the caregiver's own needs or rest [10, 42, 44]. Many families struggle to navigate the healthcare system or access needed services [19]. The isolation that comes from caregiving responsibilities can further exacerbate these challenges, as caregivers might feel cut off from social interactions and support networks.

Confronting these challenges head‐on, Nigeria can pave the way for a future where dementia care is accessible, compassionate, and culturally competent, thereby improving the quality of life for individuals with dementia and their families.

### Milestones on national dementia plan in Nigeria

There is no direct references to specific policies or milestones for dementia care in Nigeria and many African countries, Table 1 is offered the types of initiatives typically involved in addressing dementia in Nigeria. The absence of detailed milestones and policies is an important data gap in understanding the epidemiology, care needs, and effective interventions for dementia in Nigeria and similar contexts.

**TABLE 1:** Milestone on Dementia in Nigeria.

Year	Key policies or actions adopted
Pre‐2021	Epidemiological studies looking at prevalence of dementia in Nigeria [14, 30] Awareness and education programs launched to improve understanding of dementia National Ageing Policy [22]
2021	Initiation of dementia research consortia to focus on epidemiological, genetic, and clinical studies of dementia [4]
Ongoing	Continuous training for healthcare professionals on care of older adults in Nigeria [45]
Future direction	Development of national guidelines for diagnosing, treating, and caring persons with dementia in Nigeria
Future directions	Establishment of community support programs to assist families and caregivers of dementia patients. Collaboration with traditional/indigenous/spiritual healing centers to support people with dementia and their families in Nigeria
Future directions	Expansion of dementia research to include more genetic studies and the development of culturally sensitive diagnostic tools in Nigeria
Future directions	Implementing national dementia care plans or policies, integrating health and social care services in Nigeria

### Feasibility and applicability of the WHO global dementia action plan in Nigeria

As discussed earlier, the WHO Global Action Plan on Dementia aims to improve the lives of people with dementia, their carers, and families, while decreasing the impact of dementia on communities and countries [18]. The plan focuses on several areas including raising awareness about dementia, reducing the risk of developing dementia, making it easier to get a diagnosis, improving care and treatment for people with dementia, supporting carers, and increasing research.

**FIGURE 1:**
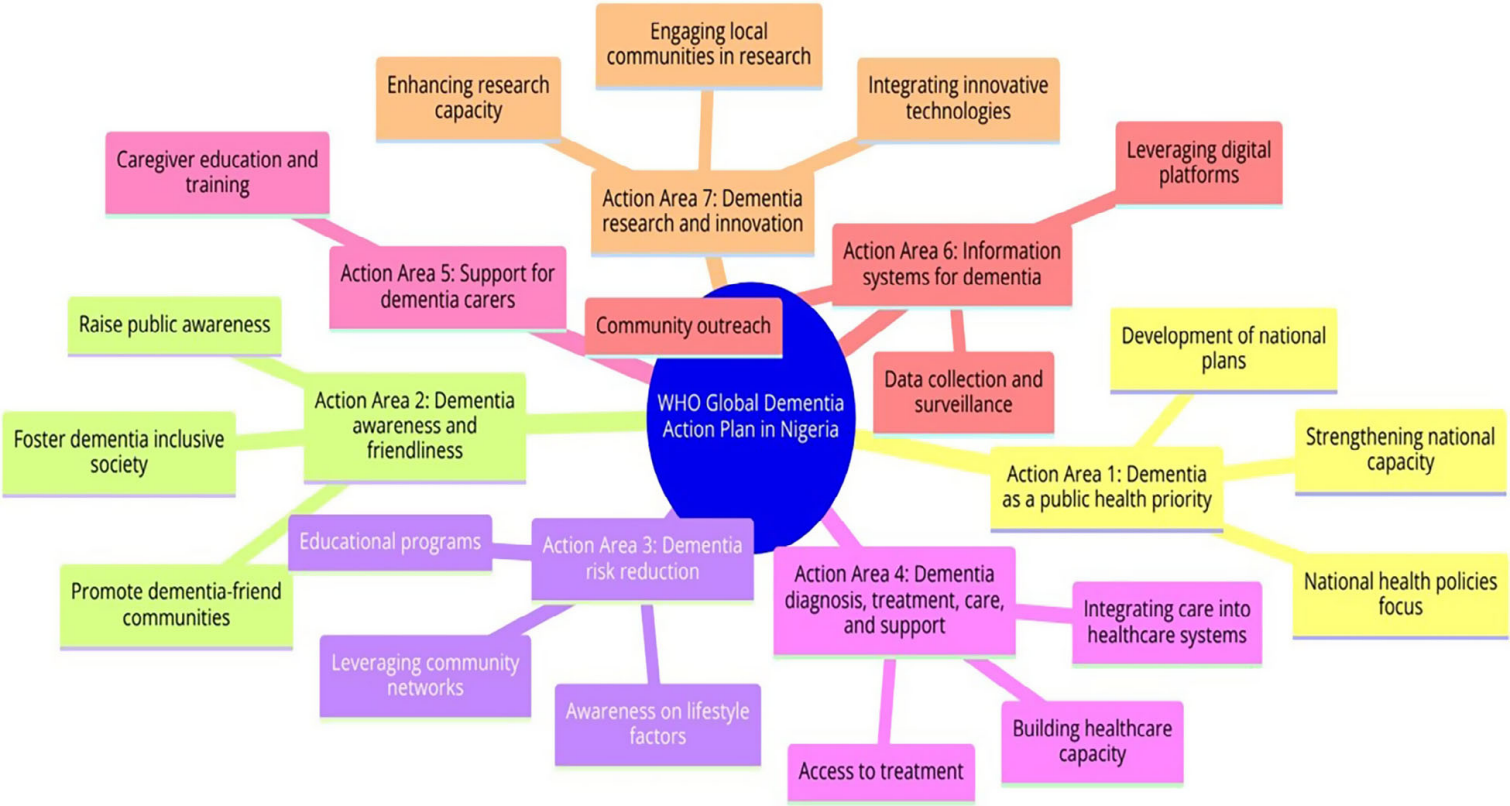
Global action plan on public health response to dementia for Nigeria.

In terms of applicability and feasibility, the WHO's global action plan could serve as a comprehensive framework for Nigeria to develop its own national dementia plan. The plan could be tailored to address the country's unique cultural and demographic needs. It would involve promoting public awareness of dementia, improving healthcare quality, social care, and long‐term care support and services for people with dementia and their families. However, the implementation of such a plan would require commitment and resources from various stakeholders, including the government, healthcare providers, and the community. It would also be crucial to involve people living with dementia and their families in the planning and implementation process to ensure that their needs and perspectives are considered (see Figure 1).

Detailed applicability and feasibility of these action plans in Nigeria are discussed as follows:

*Action area 1 (Dementia as a public health priority)*: In Nigeria, there is an emphasis on the burden of dementia: Dementia incidences are expected to increase on an exponential rate; caregiving strain, stigma, limited awareness or understanding of dementia, and economic impact, influenced by the increasing population aging and the changes in lifestyle. The main objectives of this action include: first, increasing the focus on dementia in national health policies, with the aim of making dementia a national health priority, second, to develop, implement, monitor, and evaluate national dementia plans and policies in the countries, and third, to strengthen national capacity for better understanding about dementia [18].
  Although these objectives are achievable in Nigeria, out‐of‐pocket costs continue to be the most popular method of funding medical care, making up more than 70% of total costs between 2005 and 2022 [46, 47]. Still, the current health spending as a proportion of GDP varied between 3.81% and 3.65% throughout that same time frame. Additionally, from 2005 to 2022, the domestic general government's health spending as a percentage of GDP varied between 0.68% and 0.83%, whereas the federal government's expenditure as a percentage of current health expenditure varied between 13% and 23% during the same time [48]. These highlight that the organization of healthcare for people with dementia is loose and fragmented. Of particular interest is the primary healthcare system, which might be an important tool for ensuring the feasibility of a national dementia plan, unfortunately, it is incapable of meeting the complex challenges that are implied for a more adequate healthcare deliver for people with dementia in Nigeria.
*Action area 2 (Dementia awareness and friendliness)*: This is the second action area, which emphasizes raising public awareness about dementia and creating a dementia‐friendly society. Key objectives include: first, to raise public awareness and understanding of dementia to reduce stigma and discrimination, second, to foster a dementia‐inclusive society, respect for the rights of people with dementia and support for their carers, and third, to promote dementia‐friend communities and organizations.
  The applicability of this action area in Nigeria is the enhancement of the healthcare professionals’ skills to provide quality care for individuals with dementia. In Nigeria, investing in training programs for healthcare workers, caregivers, and community leaders can contribute to building a knowledgeable and supportive environment for those affected by dementia. The plan emphasizes the importance of involving communities in dementia‐related initiatives. In Nigeria, where communal ties are strong, leveraging community networks can enhance awareness and understanding of dementia. Local leaders and influencers can play a pivotal role in disseminating information and dispelling dementia myths.
  In a bid to make this action area feasible, the Nigerian government need financial resources. Mobilizing funds through public‐private partnerships, international collaborations, and government allocations can support the establishment of dementia‐friendly services and infrastructure. Adapting global strategies to the local context is crucial. The plan should be sensitive to Nigeria's diverse cultures and languages to ensure that awareness campaigns and educational materials are culturally relevant and accessible to all segments of the population. With strategic resource mobilization, capacity building, and multi‐sectoral collaboration, Nigeria can create a dementia‐friendly environment, even without a specific national strategy. Adopting and adapting the WHO plan can pave the way for improved dementia awareness, support, and care in Nigeria.
*Action area 3: Dementia risk reduction Action area*: The applicability of this important action area is that it emphasizes the importance of raising awareness about lifestyle factors contributing to dementia. Adapting global awareness campaigns to Nigeria's cultural context can promote understanding of risk factors such as physical inactivity, smoking, and unhealthy diets, encouraging individuals to adopt healthier lifestyles [49]. Implementing educational programs targeted at various age groups can empower Nigerians with knowledge about dementia risk reduction. Incorporating preventive measures into school curricula, and community health initiatives can be instrumental in fostering a culture of proactive brain health. Leveraging community networks to disseminate information on dementia risk reduction is essential. Local leaders, religious institutions, and community organizations can serve as conduits for health messages, fostering a sense of collective responsibility toward adopting and promoting healthy lifestyles.
  Although Nigeria may face resource constraints, prioritizing dementia risk reduction within existing health budgets can be feasible. Emphasizing cost‐effective interventions, such as community‐based awareness campaigns and educational initiatives, can maximize impact within limited resources. Engaging with private sector entities, nongovernmental organizations, and international partners can bolster the implementation of dementia risk reduction strategies. Collaborations can bring in additional resources, expertise, and support to enhance the reach and effectiveness of preventive programs. Embedding dementia risk reduction strategies within existing health infrastructure is crucial for sustainability. Integrating preventive measures into routine healthcare services, leveraging primary healthcare centers, and utilizing community health workers can optimize resources and ensure widespread coverage. Therefore, despite the absence of a national strategy on dementia, Nigeria has the potential to proactively address dementia risk reduction through a comprehensive, culturally sensitive approach, aligning with the global framework outlined by the WHO.
*Action area 4: Dementia diagnosis, treatment, care, and support*: This plan emphasizes the importance of early and accurate diagnosis. In Nigeria, there is a need to build capacity for healthcare professionals to recognize and diagnose dementia. Training programs, workshops, and awareness campaigns can be implemented to enhance diagnostic capabilities. Collaborations with international partners can facilitate the exchange of expertise and best practices in dementia diagnosis. Access to appropriate treatment is crucial in managing dementia effectively. This provides evidence‐based and affordable treatment options. Nigeria can explore partnerships with pharmaceutical companies, international organizations, and research institutions to ensure the availability and affordability of dementia medications. Additionally, integrating dementia care into existing primary healthcare systems can enhance treatment accessibility nationwide.
  The quality of care for individuals with dementia and supporting their caregivers is still a significant challenge. In Nigeria, informal caregiving is prevalent, and it is essential to develop training programs for caregivers. This can include psychosocial support, respite services, and educational resources. Community‐based care models can be implemented, leveraging existing structures such as community health workers and traditional healers. Although the implementation of the WHO Global Dementia Action Plan in Nigeria is crucial, some challenges must be considered. These include limited healthcare infrastructure, resource constraints, and cultural factors influencing healthcare‐seeking behavior [4, 50]. However, leveraging existing healthcare platforms, engaging local communities, and integrating dementia care into broader health initiatives can enhance feasibility. In Nigeria, where there is currently no national strategy on dementia, adopting and tailoring the WHO Action Plan can significantly improve diagnosis, treatment, care, and support. Collaborative efforts involving government agencies, healthcare professionals, international partners, and local communities are vital for successfully implementing this plan in the Nigerian context.
*Action area 5: Support for dementia carers*: Caregivers play a pivotal role in managing the day‐to‐day challenges of individuals with dementia [13]. The WHO Action Plan recognizes the importance of providing support to caregivers to enhance the quality of care provided. One key aspect of the WHO Action Plan is the emphasis on caregiver education and training. In Nigeria, where awareness about dementia is limited, establishing training programs can empower caregivers with the necessary skills and knowledge. Collaboration with local healthcare institutions and nongovernmental organizations can facilitate the dissemination of information and support networks for caregivers. Given the familial structure prevalent in Nigeria, community‐based support systems can be leveraged to assist dementia caregivers. The WHO Action Plan encourages the development of local support groups and networks. Establishing such community‐focused initiatives can alleviate the isolation often experienced by caregivers and foster a sense of shared responsibility. Caregivers in Nigeria often face financial challenges and limited access to social services. However, the WHO Action Plan recommends economic support mechanisms for caregivers, acknowledging the financial strain associated with dementia care. Still, tailoring these recommendations to the Nigerian context involves collaboration between government agencies and nonprofit organizations to establish targeted financial aid programs [38]. The feasibility of implementing caregiver support initiatives is contingent on addressing challenges such as limited resources, cultural stigmas surrounding dementia, and the need for a nuanced understanding of regional diversity. However, existing healthcare structures, community engagement strategies, and international collaborations present opportunities to overcome these challenges.
*Action area 6: Information systems for dementia*: In Nigeria, where awareness and understanding of dementia are limited, the development of information systems can serve as a cornerstone for establishing a comprehensive national strategy. Creating awareness about dementia is paramount in Nigeria, where misconceptions and stigmas surround cognitive disorders. Leveraging information systems, such as digital platforms and community outreach programs, can disseminate accurate information, raise public awareness, and reduce the social stigma associated with dementia. Establishing robust information systems enables efficient data collection and surveillance, crucial for understanding the prevalence, risk factors, and trends of dementia in Nigeria. Collaborative efforts with healthcare institutions, research organizations, and governmental bodies can facilitate the integration of dementia‐related data into existing health information systems. Given Nigeria's vast and diverse population, incorporating telemedicine and remote support systems into information networks can bridge gaps in access to dementia care. Telehealth initiatives can facilitate remote consultations, caregiver support, and education, extending the reach of dementia‐related services to underserved regions. While implementing information systems for dementia in Nigeria poses challenges such as limited technological infrastructure and digital literacy, there are opportunities to capitalize on the widespread use of mobile phones and community engagement strategies. Public–private partnerships, international collaborations, and capacity‐building initiatives can address these challenges.
*Action area 7: Dementia research and innovation*: In Nigeria, fostering a research environment can enhance knowledge about the prevalence, risk factors, and cultural dimensions of dementia. Research‐driven insights are essential for formulating evidence‐based policies and interventions. Building research capacity in Nigeria involves establishing collaborations among research institutions, healthcare facilities, and international partners [4]. Strengthening academic and clinical research networks can create an ecosystem conducive to generating knowledge on dementia, fostering innovation, and developing locally relevant solutions. Incorporating innovative technologies for dementia diagnosis and treatment is crucial. The WHO Action Plan emphasizes adopting innovative approaches to enhance diagnostic accuracy and treatment efficacy. Nigeria can leverage partnerships with technology companies, research institutions, and healthcare providers to integrate innovative solutions into dementia care. Engaging local communities in dementia research is vital for understanding cultural nuances and tailoring interventions. Community‐based participatory research can enhance the relevance and acceptance of research findings, fostering a sense of ownership and collaboration in addressing dementia‐related challenges. Feasibility considerations involve the establishment of research infrastructures, including dedicated dementia research centers, data repositories, and ethical frameworks. Collaborations with international organizations and funding agencies can provide resources and expertise to support the development of research infrastructure in Nigeria. The WHO Global Dementia Action Plan provides a robust framework for advancing dementia research and innovation in Nigeria; hence, aligning strategies to the local context, building research capacity, and addressing ethical considerations are essential for successfully implementing research‐driven interventions. Collaborative efforts involving government agencies, academic institutions, healthcare providers, and the public are crucial for establishing a sustainable and impactful dementia research ecosystem in Nigeria.

In all, this article underscores the relevance of the WHO Global Dementia Action Plan in Nigeria, offering holistic strategies for considering dementia as a public health priority, awareness and friendliness with people with dementia, risk reduction, diagnosis, treatment, care and support, caregiver support, information systems development, and dementia research and innovation. The success of these initiatives is contingent upon collaborative efforts involving government bodies, healthcare institutions, technology providers, research organizations, and local communities. Adopting and adapting the WHO Action Plan within the Nigerian context can pave the way for a comprehensive and sustainable approach to dementia caregiving in Nigeri

## RECOMMENDATIONS

A national dementia strategy involves a multi‐sectoral approach to implementation that engages individuals with dementia, their families, and government and nongovernment agencies. Nigeria's demographic shift presents challenges and opportunities for the nation, and planning theory offers valuable insights into the formulation and execution of an effective strategy. The combination of an aging population and the rising prevalence of dementia demands a proactive and comprehensive approach to address the multifaceted issues associated with this condition. By adopting a planning theory perspective, Nigeria can better navigate the complexities of its demographic changes and design a sustainable and inclusive dementia strategy. Therefore, an advocacy that moves on to the development and implementation of a national dementia strategy that aligns with the WHO Global Dementia Action Plan is important. This framework should prioritize dementia as a public health issue and foster an environment of inclusivity and support for affected individuals and their families.

There is a need for nationwide campaigns to destigmatize dementia, emphasizing its medical basis and dispelling myths. To foster understanding and empathy, educational initiatives should target all societal levels, from policymakers to grassroots communities. Invest in the expansion and training of healthcare professionals in dementia care. This includes establishing specialized care facilities, integrating dementia care into primary healthcare settings, and leveraging technology for remote care solutions. There is need for more research funding aimed at understanding dementia's sociocultural dynamics in Nigeria, exploring innovative care and treatment methods, and evaluating the effectiveness of policy implementations. Development of community‐based programs enables individuals with dementia to maintain their dignity and quality of life. These programs should facilitate access to care, social engagement, and support for families and caregivers.

Implement financial aid schemes to alleviate the economic burden on families caring for relatives with dementia. This could include subsidies for medications, care services, and support for caregivers. A robust system monitors and evaluates the impact of the national dementia strategy. This should involve regular assessments to ensure that the strategy remains relevant, effective, and responsive to the population's evolving needs.

## CONCLUSION

Nigeria faces an escalating dementia crisis driven by a growing ageing population and lack of a national strategies for dementia care. Challenges include inadequate healthcare infrastructure, stigma, and the absence of a tailored national dementia strategy aligned with global best practices. Despite these, a paradigm shift toward an inclusive, culturally appropriate, and sustainable approach is important. Exploring Nigeria's readiness for implementing a dementia strategy reveals opportunities amidst challenges. The WHO's Global Dementia Action Plan offers a pragmatic framework, and success hinges on stakeholder commitment in Nigeria. Dementia must be recognized as a health issue and a societal challenge requiring a collaborative, multi‐sectoral approach involving government, healthcare providers, and communities. Navigating cultural beliefs, healthcare limitations, and economic constraints, there is a compelling argument for concerted efforts to raise awareness, build healthcare capacities, and foster research and innovation. The envisaged national dementia strategy holds promise as a comprehensive blueprint to enhance the quality of life for individuals with dementia and their caregivers, positioning Nigeria as a leader in dementia care on the African continent. A unified response is imperative to address the forthcoming surge of dementia. This response must transcend policy formulation to prioritize dementia as a holistic public health priority.

## AUTHOR CONTRIBUTIONS

*Conceptualization; investigation; funding acquisition; writing—original draft; writing—review and editing; resources*: Oluwagbemiga Oyinlola.

## CONFLICT OF INTEREST STATEMENT

The author declares no conflicts of interest.

## FUNDING INFORMATION

No funding was received for this research.

## ETHICS STATEMENT

Not applicable.

## Data Availability

The data supporting this study's findings are available from the corresponding author upon reasonable request.
